# Uncovering the Metabolic Origins of Polycystic Ovary Syndrome

**DOI:** 10.7759/cureus.114165

**Published:** 2026-08-08

**Authors:** Pracruti J Iyer, Hasim Reza, Nithisha Chitteti, Anjali Singh, Rishabh Goyal, Sajidali S Saiyad, Aishwarya Emima Raj Stephen Raj, Navya Singla, Bhavya Vats, Dharssini Kamaladasan, Princejeet Singh Chahal, Ashesh Das, Pavani Reddy, Alisha Lakhani

**Affiliations:** 1 Medicine and Surgery, BKL Walawalkar Rural Medical College, Chiplun, IND; 2 Medicine, Central America Health Sciences University, Belize, BLZ; 3 Obstetrics and Gynaecology, Maternity Hospital, Hyderabad, IND; 4 Dentistry, King George's Medical University, Lucknow, IND; 5 Internal Medicine, Kasturba Medical College, Manipal, Udupi, IND; 6 Physiology, Pacific Medical College and Hospital, Pacific Medical University, Udaipur, IND; 7 Medicine, Nicolae Testemițanu State University of Medicine and Pharmacy, Chisinau, MDA; 8 Medicine, Mahatma Gandhi Mission (MGM) Medical College, Mumbai, IND; 9 Medicine, DY Patil University, Mumbai, IND; 10 Family Medicine, Coimbatore Medical College, Coimbatore, IND; 11 Medicine, Research MD, Vadodara, IND; 12 Internal Medicine, KPC Medical College and Hospital, Kolkata, IND; 13 Medicine, Shantabaa Medical College, Amreli, IND

**Keywords:** cardiometabolic risk, glp-1 receptor agonists, homa-ir, hyperandrogenism, infertility, insulin resistance, metabolic origins, pmos, polycystic ovary syndrome, polyendocrine metabolic ovarian syndrome

## Abstract

Polycystic ovary syndrome (PCOS), now known as polyendocrine metabolic ovarian syndrome (PMOS), though the two terms remain in concurrent use during the ongoing transition, is a common endocrine disorder affecting women of reproductive age, characterized by disrupted hormonal balance and impaired metabolic health. Insulin resistance (IR) is a central pathological feature of PCOS, contributing to a broad spectrum of metabolic, reproductive, and psychological complications. Resulting hyperinsulinemia worsens hyperandrogenism, driving acne, hirsutism, menstrual irregularities, and infertility, while also impairing follicular development and ovulation. Because IR is closely tied to obesity, it further elevates the risk of type 2 diabetes and cardiovascular disease, compounding the long-term health burden of PCOS. This review examines the association between IR and PCOS, with particular attention to its influence on clinical presentation, diagnostic approaches, treatment strategies, and disease progression. A clear understanding of IR's role is essential for effective management, as targeting it through lifestyle modification and pharmacological intervention can improve reproductive outcomes and reduce long-term metabolic and cardiovascular risk in women with PCOS.

## Introduction and background

Polycystic ovary syndrome (PCOS) is a multifactorial endocrine disorder affecting women of reproductive age, typically between 18 and 44 years. The reported prevalence varies according to the diagnostic criteria used and the population studied, with studies applying the Rotterdam criteria generally reporting higher prevalence estimates than other diagnostic definitions [1]. According to the Rotterdam criteria, PCOS is diagnosed by the presence of at least two of the following three features after exclusion of related disorders: clinical and/or biochemical hyperandrogenism, ovulatory dysfunction, and polycystic ovarian morphology on ultrasonography. Anti-Müllerian hormone (AMH) has also emerged as a useful adjunctive biomarker for defining polycystic ovarian morphology in selected adult women, although it complements rather than replaces the established diagnostic criteria [1]. In May 2026, a global multistep consensus process spanning 56 patient and professional organizations formally renamed the condition polyendocrine metabolic ovarian syndrome (PMOS), a change intended to better reflect the systemic endocrine and metabolic manifestations of the disorder rather than emphasizing ovarian morphology alone. The diagnostic criteria and clinical management remain unchanged, and adoption of the new terminology is expected to proceed gradually [2]. As the literature reviewed here predates this nomenclature change, the established term PCOS is retained throughout this review, with the understanding that it is synonymous with PMOS. Chronic hormonal dysregulation in PCOS can lead to menstrual irregularities, anovulation, infertility, and characteristic polycystic ovarian morphology [3].

PCOS is associated with several metabolic and reproductive comorbidities, including insulin resistance (IR), obesity, dyslipidemia, type 2 diabetes mellitus, psychological disorders, and cardiovascular disease. Although its precise etiology remains incompletely understood, genetic susceptibility, environmental factors, physical inactivity, and unhealthy dietary habits are believed to contribute. The pathogenesis involves a complex interplay between intrinsic IR, visceral adiposity, hyperinsulinemia, and neuroendocrine dysregulation, which together promote hyperandrogenism and metabolic dysfunction. The diagnosis of PCOS is established using the Rotterdam criteria through a combination of clinical evaluation, biochemical assessment, and ultrasonographic findings after excluding other disorders that may mimic the syndrome. Management is individualized and focuses on alleviating hyperandrogenic symptoms, restoring menstrual regularity and fertility when desired, and reducing long-term metabolic risk [4].

IR refers to a reduced ability of both endogenous and exogenous insulin to stimulate glucose uptake and utilization compared with healthy individuals [5]. Elevated androgen levels affect granulosa cell function and follicular development, contributing to obesity and IR, while obesity further exacerbates IR, creating a self-perpetuating cycle of metabolic and reproductive dysfunction [6]. IR is estimated to affect approximately 50-80% of women with PCOS and is generally more pronounced in severe phenotypes, where compensatory hyperinsulinemia enhances ovarian androgen production, disrupts gonadotropin regulation, and contributes to disease progression [6].

This review aims to comprehensively summarize the current understanding of IR in PCOS, with particular emphasis on its pathophysiological mechanisms, clinical manifestations, diagnostic approaches, and current as well as emerging therapeutic strategies.

## Review

Methods

This narrative review was conducted by searching PubMed/MEDLINE, Scopus, Google Scholar, and Web of Science for English-language articles published up to May 2026. Search terms included *"polycystic ovary syndrome" OR "PCOS" OR "polyendocrine metabolic ovarian syndrome" OR "PMOS" AND "insulin resistance", "pathophysiology", "diagnosis", "treatment", "GLP-1 receptor agonists", "metformin", "HOMA-IR", "obesity", and "lifestyle intervention". Original research articles, systematic reviews, meta-analyses, international clinical practice guidelines, and high-quality narrative reviews relevant to insulin resistance in PCOS were considered. Conference abstracts, non-English publications, duplicate records, animal-only studies, and articles lacking sufficient methodological detail were excluded. Relevant information regarding study characteristics, principal findings, and clinical implications was extracted manually and synthesized narratively.

As this is a narrative review, no formal risk-of-bias assessment or quantitative synthesis was performed.

Pathophysiology of insulin resistance in PCOS

Insulin plays a central role in glucose homeostasis by binding to its cell-surface receptor and activating the phosphatidylinositol 3-kinase (PI3K)/Akt signaling pathway, which promotes GLUT4 translocation to the plasma membrane and facilitates glucose uptake in insulin-sensitive tissues. In parallel, activation of the mitogen-activated protein kinase (MAPK)/ERK pathway primarily regulates cell growth, proliferation, and differentiation rather than glucose transport [7]. Beyond impaired insulin signaling, chronic low-grade inflammation, oxidative stress, mitochondrial dysfunction, and adipokine imbalance further contribute to insulin resistance in PCOS. Dysregulated secretion of adipokines such as adiponectin and leptin from visceral adipose tissue disrupts metabolic homeostasis, while inflammatory cytokines including TNF-α and IL-6 impair insulin signaling pathways, promoting both metabolic dysfunction and hyperandrogenism [8]. The neuroendocrine-adipose axis also plays a central role in disease pathogenesis through interactions between adipose-derived mediators, hypothalamic-pituitary signaling, and ovarian steroidogenesis. Hyperinsulinemia amplifies luteinizing hormone-mediated androgen production, whereas adipose tissue dysfunction perpetuates systemic inflammation and insulin resistance, creating a self-reinforcing pathogenic cycle [8].

The proinsulin-to-insulin ratio can serve as a useful marker of IR and beta-cell function; overweight or obese women with PCOS often show increased insulin secretion and elevated proinsulin levels, contributing to hyperinsulinemia [9].

PCOS also has a strong hereditary component. Hyperinsulinemia can develop early in life and persist through puberty in girls with a family history of PCOS, even before a formal diagnosis, potentially linked to impaired pancreatic beta-cell function [6]. Increased activity of the SH2 domain-containing adaptor protein in ovarian cells may suppress insulin signaling and contribute to IR, and skin fibroblasts from women with PCOS also show intrinsic insulin resistance, although genetic studies have ruled out insulin receptor gene mutations as a primary cause [7].

Clinical manifestations of IR in PCOS

Weight Gain and Obesity

PCOS is commonly associated with obesity, which adversely affects reproductive health and is linked to higher circulating androgens and insulin, worsening PCOS symptoms [9]. Women with PCOS and obesity exhibit more pronounced hyperandrogenism and IR; obesity-related chronic inflammation further impairs ovarian function by reducing insulin sensitivity, establishing a self-reinforcing cycle [10].

Type 2 Diabetes Risk

IR contributes to abnormalities in glucose metabolism and lipid profiles, raising the long-term risk of type 2 diabetes [11]. Compensatory hyperinsulinemia drives many PCOS manifestations, including androgen excess, acne, hirsutism, and reproductive irregularities; if beta-cell function later deteriorates, the reduced compensatory capacity can progress to glucose intolerance and overt type 2 diabetes [11].

Acanthosis Nigricans

Thickened, dark, velvety skin at the neck, underarms, and groin is an early clinical indicator of IR. Elevated insulin can activate insulin-like growth factor receptor 1 (IGF1R) in skin and ovarian tissue, contributing to these dermatological changes, along with skin tags in friction-prone areas, particularly in obese individuals [12].

Ovarian Dysfunction and Infertility

Most women with PCOS experience ovarian dysfunction: 70-80% show oligomenorrhea or amenorrhea, and PCOS is the most common medically manageable cause of infertility, accounting for roughly 70% of anovulatory cases [13]. Affected women face elevated risk of miscarriage and pregnancy complications such as gestational diabetes, hypertension, and preeclampsia, and about 60% experience increased time to conceive [13].

Psychological Symptoms

PCOS significantly affects quality of life and psychological well-being, with associated psychiatric comorbidities, neurocognitive impairment, and sexual dysfunction. 

Women with PCOS face increased risk of mood and anxiety disorders and report higher depression levels independent of BMI [14].

Cardiovascular Implications

Although findings are somewhat inconsistent, most studies link PCOS to elevated cardiovascular risk, including coronary heart disease and stroke, independent of BMI. IR drives cardiometabolic abnormalities such as dyslipidemia, hypertension, and impaired glucose regulation, and women with PCOS more often show subclinical markers like elevated coronary calcium scores, C-reactive protein, and carotid intima-media thickness [15].

The heterogeneous clinical presentation of PCOS encompasses reproductive, endocrine, metabolic, and psychological features, as summarized in Table 1.

**Table 1:** Phenotypic Heterogeneity and Clinical Markers in PCOS/PMOS A comprehensive overview of the reproductive, endocrine, metabolic, and neuro-psychological features defining polyendocrine metabolic ovarian syndrome (formerly polycystic ovary syndrome). Table created using information from ref [10-15] PCOS: Polycystic ovary syndrome; PMOS: polyendocrine metabolic ovarian syndrome

Category	Manifestations	Description
Reproductive Features	Oligomenorrhea/amenorrhea	Infrequent (<8/year) or absent menstrual periods
	Anovulation/oligoovulation	Absent or irregular ovulation, contributing to infertility
	Polycystic ovarian morphology	Enlarged ovaries with multiple small follicles on ultrasound
	Pregnancy-related complications	Increased risk of miscarriage, gestational diabetes, preeclampsia
Endocrine Features	Hirsutism	Coarse terminal hair growth in androgen-dependent areas
	Acne	Comedonal and nodular acne, often on face, chest, back
	Androgenic alopecia	Patterned hair thinning at crown/temples
	Acanthosis nigricans	Darkened, velvety skin at neck, underarms, groin
Metabolic Features	Insulin resistance	Reduced insulin sensitivity, often with hyperinsulinemia
	Type 2 diabetes/dyslipidemia	Impaired glucose and lipid metabolism, elevated cardiometabolic risk
	Obesity	Weight gain, especially central/abdominal adiposity
Psychological Features	Mood disorders	Anxiety, depression, body-image concerns, reduced quality of life

Diagnostic tools for IR in PCOS

Insulin Tolerance Test

The short insulin tolerance test (SITT) estimates IR by giving intravenous insulin and sampling blood at close intervals over 15 minutes; the resulting K Index (KITT) reflects the rate of plasma glucose decline and indicates insulin sensitivity [16]. While the SITT can assess whole-body insulin sensitivity even in patients on insulin therapy, it carries hypoglycemia risk and requires multiple samples. A subcutaneous variant avoids venous sampling and lowers hypoglycemia risk, though its accuracy is not yet well validated against the intravenous method [17]. Using the intravenous KITT, women with PCOS show a higher prevalence of IR than controls, with amenorrheic women more affected than those with oligomenorrhea; IR has been identified in up to 80% of women with PCOS using this method [17].

HOMA-IR (Homeostatic Model Assessment)

HOMA-IR is a simple, non-invasive tool widely used to estimate IR and beta-cell function from fasting glucose and insulin levels, calculated as fasting insulin (mU/L) × fasting glucose (mmol/L) ÷ 22.5 [18]. Although HOMA-IR is widely used to estimate insulin resistance, no universally accepted diagnostic cut-off exists. Threshold values vary across populations, ethnicities, and assay methodologies [18]. Elevated HOMA-IR is seen in both lean and obese women with PCOS but is more pronounced with obesity [18]. Given its simplicity, HOMA-IR may serve as an adequate substitute for more invasive testing methods.

Fasting Insulin and Glucose Levels

Fasting insulin and glucose measurements are commonly used to estimate IR, though their predictive accuracy for abnormal glucose tolerance is relatively limited [19]. Fasting insulin correlates positively with cardiovascular and metabolic risk in women with PCOS, and the fasting glucose-to-insulin ratio has shown the strongest correlation with insulin sensitivity, outperforming fasting insulin or glucose alone as a single screening tool (sensitivity 95%, specificity 84%) [20].

OGTT (Oral Glucose Tolerance Test)

The OGTT measures blood glucose at set intervals after a 75g oral glucose load and is a standard tool for detecting glucose intolerance or type 2 diabetes, though it does not directly assess insulin sensitivity [21]. Per American Diabetes Association criteria, prediabetes is defined by fasting glucose 100-125 mg/dL, two-hour glucose 140-199 mg/dL, or HbA1c 5.7-6.4%, with type 2 diabetes diagnosed above these thresholds [21]. The 2023 International PCOS Guideline identifies OGTT as the most reliable screening method for type 2 diabetes in women with PCOS, outperforming fasting glucose or HbA1c alone [22].

Therapeutic approaches to managing IR in PCOS

Lifestyle Modifications

Lifestyle modification is the first-line management strategy for PCOS, particularly for women presenting with infertility [22]. Dietary change and exercise address IR and improve ovulation, menstrual regularity, weight, and psychological well-being, though strict calorie restriction alone often fails to produce lasting benefit [23].

Exercise: Current international guidelines recommend regular aerobic and resistance exercise as part of first-line management for PCOS, with exercise prescriptions individualized according to patient characteristics and treatment goals [24]. High-intensity interval training (HIIT) for 12-24 weeks has been shown to improve insulin sensitivity, body fat, LDL cholesterol, and inflammatory markers, with guidelines recommending at least 90 minutes of HIIT weekly. Yoga has also been shown to reduce stress and obesity while improving hormonal balance and insulin sensitivity [25].

Dietary intervention is a cornerstone of PCOS management, with current guidelines recommending individualized, sustainable dietary patterns rather than a single specific diet. In overweight or obese women, a modest weight loss of 5-10% can significantly improve insulin sensitivity, ovulatory function, menstrual regularity, and metabolic health [23]. Diets emphasizing whole grains, fruits, vegetables, legumes, lean protein, and unsaturated fats while limiting refined carbohydrates, sugar-sweetened beverages, and saturated fats have demonstrated beneficial effects on insulin resistance and cardiovascular risk factors [23,24]. Low-glycemic-index diets and Mediterranean or DASH-style dietary patterns have been associated with improved glycemic control, lipid profiles, and reproductive outcomes in women with PCOS [25,26]. Dietary management should be individualized and combined with regular physical activity to optimize long-term metabolic and reproductive outcomes [23,24].

Sleep and mood modification: Sleep disturbances are common among women with PCOS and negatively affect quality of life, mood, and stress, though clinical research on targeted interventions remains scarce [25]. Shared factors such as androgen excess, IR, and obesity may link PCOS with mood disorders, and screening for these issues may improve overall well-being [25].

Pharmacological treatments

International PCOS guidelines recommend medical therapy as a second-line approach-after lifestyle modification-for irregular cycles, hirsutism, and anovulation, given hyperinsulinemia's role in driving hyperandrogenism [27]. Commonly used agents include insulin sensitizers (metformin), anti-obesity agents, combined oral contraceptive pills (COCPs), and anti-androgens [28].

Metformin

Metformin, an insulin-sensitizing anti-diabetic agent, is considered the cornerstone of metabolic therapy in PCOS, reducing hepatic glucose production and improving insulin sensitivity, with additional benefit in preventing late miscarriage and preterm birth in pregnant women with PCOS [29]. It improves menstrual regularity, hyperinsulinemia, and hyperandrogenism, and may help prevent long-term cardiovascular complications, though as monotherapy it produces only mild-to-moderate improvement and is generally less effective than COCPs; current guidelines therefore favor combining metformin with COCPs in overweight or obese women, including adolescents [27]. Gastrointestinal side effects (nausea, diarrhea, flatulence) are common [30].

Thiazolidinediones (TZDs)

TZDs improve hyperglycemia and dyslipidemia and reduce hepatic and peripheral IR by activating PPARγ, also helping regulate menstrual cycles and reduce androgen levels. However, they carry risks of weight gain, peripheral edema, and heart failure. Combination therapy with metformin has shown greater benefit for menstrual regularity and HOMA-IR than either agent alone [31].

Inositol

Inositol supplementation has emerged as a potential alternative to metformin, acting as an insulin sensitizer that supports menstrual regularity and reduces hyperandrogenism. Myo-inositol and D-chiro-inositol, its two main isomers, respectively improve ovulatory function and reduce peripheral IR, though evidence remains insufficient for inositol to be recommended as standard therapy [30].

Combined Oral Contraceptive Pills

COCPs are widely used to manage irregular cycles and hyperandrogenic symptoms such as hirsutism and acne and are recommended for both adult and adolescent women with PCOS, including those at risk of the condition [28]. They reduce free testosterone by stimulating hepatic sex hormone-binding globulin (SHBG) production, though estrogen components-particularly ethinyl estradiol-increase venous thromboembolism risk from roughly 2-10 to 7-10 events per 10,000 woman-years. Later-generation progestins raise SHBG more than second-generation formulations and may have modest, clinically relevant effects on lipid profiles [31].

Anti-androgen Medications

Anti-androgens such as spironolactone, flutamide, finasteride, and cyproterone acetate may help relieve hyperandrogenic symptoms, though the 2018 PCOS guidelines note insufficient evidence to recommend their routine use [28].

New and emerging treatments

GLP-1 receptor agonists (GLP-1RAs), including liraglutide, tirzepatide, dulaglutide, and semaglutide, have emerged as effective adjunctive therapies for weight management and metabolic complications in women with PCOS who have overweight or obesity, particularly when lifestyle interventions alone are insufficient. However, they are not recommended as routine first-line therapy for all women with PCOS, and treatment should be individualized according to current clinical guidelines [32]. In a trial of 150 women with PCOS and impaired glucose tolerance, prediabetes remission rates were 64% with combination therapy versus 56% with exenatide alone and 32% with metformin alone [33].

Liraglutide (1.8 mg/day for 26 weeks) has produced significant reductions in body weight, liver fat, visceral adipose tissue, and free testosterone in overweight women with PCOS, and low-dose liraglutide combined with metformin has improved IVF pregnancy rates compared with metformin alone [34]. Tirzepatide improves weight and insulin sensitivity but may interact with hormonal contraception, so effective contraception and a two-to-three month washout period are advised before attempting conception [35]. Semaglutide has produced greater weight loss than metformin or liraglutide, with roughly 80% of treated women achieving at least 5% weight loss and improved fasting glucose and HOMA-IR [36].

DPP-4 Inhibitors

Sitagliptin, the most studied DPP-4 inhibitor, blocks incretin hormone degradation to enhance glucose-dependent insulin secretion. Combination therapy with metformin has improved HOMA-IR, insulin sensitivity, and androgen indices in obese and prediabetic women with PCOS more than either agent alone, though overall clinical evidence remains inconclusive [37].

SGLT-2 Inhibitors

By enhancing insulin sensitivity and glucose excretion, SGLT-2 inhibitors improve lipid parameters and promote weight loss, including in women without diabetes [38]. Combining canagliflozin with metformin produces greater reductions in free testosterone and body weight than metformin alone, with improved menstrual regularity and lipid profiles, while dapagliflozin has improved weight, glycemic control, and HOMA-IR over a short treatment course [38]. Overall, SGLT-2 inhibitors appear to be safe agents that may aid weight loss and hormonal balance in PCOS [39].

Obesity medication, Orlistat, which is a gastric lipase inhibitor that reduces dietary fat absorption, has shown weight-loss and ovulation benefits comparable to metformin, with generally better tolerability and fewer side effects across multiple studies [40].

Role of bariatric surgery in obese patients with PCOS

Bariatric surgery is an effective treatment option for selected women with PCOS and severe obesity who meet established surgical eligibility criteria, particularly when lifestyle modification and pharmacological therapy have failed to achieve adequate weight loss or metabolic improvement. It is associated with significant improvements in weight, insulin resistance, reproductive function, and cardiometabolic health but is not considered routine therapy for all women with PCOS [41]. Candidates generally have a BMI over 40, or over 35 with two or more comorbidities; the main procedures are Roux-en-Y gastric bypass, sleeve gastrectomy, and gastric banding, usually performed laparoscopically [42].

In one study, PCOS prevalence fell from 45.6% before bariatric surgery to 6.8% one year afterward, and 71% of previously anovulatory women resumed normal cycles, with greater ovulatory recovery linked to greater weight loss [43]. The procedure is generally avoided in patients with limited life expectancy, active pregnancy or planned pregnancy, active substance use, or untreated severe psychiatric illness. Acute complications (affecting 5-10% of patients) resemble those of other abdominal surgeries, while long-term risks include nutritional deficiencies, internal hernias, and late-onset hypoglycemia; most nutritional risks can be mitigated with routine supplementation [44].

IR and PCOS: special considerations

Insulin Resistance in Lean Women with PCOS

IR is not confined to women with obesity. Non-obese women with PCOS have lower insulin levels and IR than their obese counterparts, alongside higher SHBG, but hyperinsulinemia remains common [45]. Glucose metabolism is impaired regardless of central obesity, and HOMA-IR correlates more strongly with body fat percentage in PCOS patients than in controls, suggesting PCOS-specific factors amplify lipid-related glucose impairment [45]. Lean women with PCOS carry visceral adipose tissue levels comparable to obese women, and androgens promote visceral fat accumulation and reduce adiponectin, a hormone that otherwise improves insulin sensitivity [46]. Although obesity aggravates insulin resistance, intrinsic defects in insulin signaling are also present in many lean women with PCOS, suggesting that insulin resistance represents an inherent feature of the syndrome independent of adiposity [45].

Intrauterine androgen exposure may alter PCOS-associated genes and hypothalamic-pituitary signaling, increasing LH secretion and IR risk from puberty onward. Kisspeptin neurons play a key role in this LH dysregulation, with androgens, AMH, leptin, and IR all raising the LH:FSH ratio [47]. These findings further highlight the bidirectional relationship between neuroendocrine dysfunction and insulin resistance, whereby hyperinsulinemia enhances ovarian androgen production, while androgen excess further impairs insulin sensitivity, perpetuating disease progression [47]. Lower adiponectin levels, linked more closely to IR than to testosterone, are consistently seen in women with PCOS after adjusting for BMI, and altered subcutaneous fat handling may explain the hyperleptinemia often observed in lean PCOS [48]. In one study, IR prevalence was substantial in both lean (83.3%) and overweight (93.1%) women with PCOS, indicating that IR is intrinsic to the syndrome rather than purely obesity-driven [44]. Vitamin D signaling also appears relevant to IR pathogenesis, with deficiency common among obese women with PCOS [49]. 

Stress may be an underappreciated contributor to PCOS. Elevated salivary α-amylase and cortisol, markers of sympathetic and HPA-axis activation, have been observed in overweight or obese women with PCOS relative to lean counterparts, alongside a bidirectional relationship between sympathetic activity and IR [50]. This link warrants further study, particularly in lean PCOS phenotypes.

Age and Progression of IR in PCOS

Aging influences both the presentation and severity of PCOS, largely through declining ovarian and adrenal androgen production alongside an age-related rise in IR and metabolic disturbance. Younger women with PCOS more often present with hyperandrogenism and chronic anovulation, while obesity, IR, and metabolic disorders predominate at older ages, tracking with increasing body weight over reproductive life [51]. Metabolic syndrome prevalence rises with both age and central obesity, with women over 25 with elevated waist-to-hip ratio at particular risk [51]. IR appears to follow a U-shaped pattern by age-elevated in women under 20 and over 30-independent of BMI, reflecting age-related declines in beta-cell and skeletal muscle insulin sensitivity [52]. Neuroendocrine signaling also contributes significantly to PCOS pathogenesis. Kisspeptin neurons regulate hypothalamic GnRH secretion and influence luteinizing hormone pulsatility, linking metabolic status with reproductive endocrine function. Alterations in kisspeptin signaling may contribute to hyperandrogenism, ovulatory dysfunction, and insulin resistance.

Ethnicity and genetic factors contributing to the development of PCOS

A genetic basis for PCOS is supported by twin studies showing markedly higher concordance in monozygotic than dizygotic twins, and by genome-wide association studies identifying loci shared across Han Chinese and European populations, though these account for less than 10% of PCOS heritability, implying substantial contributions from epigenetic and environmental factors [53]. Clinical presentation also varies by ethnicity: hirsutism tends to be more prevalent and severe among Middle Eastern, Mediterranean, and South Asian women than East Asian or Caucasian women, likely reflecting differences in 5-α reductase activity, supporting the use of ethnicity-specific diagnostic thresholds [54].

An evolutionary mismatch hypothesis has also been proposed, suggesting that genetic traits favoring efficient energy storage and reproductive success under ancestral environmental conditions may predispose modern women to insulin resistance and PCOS in the context of sedentary lifestyles and excess caloric intake. Although this concept remains theoretical, it provides a potential evolutionary framework for understanding disease persistence [53].

Limitations

This review has several limitations that should be acknowledged. First, the heterogeneity of diagnostic criteria for PCOS/PMOS across studies, particularly the ongoing transition from the Rotterdam criteria to the newly adopted PMOS nomenclature, may introduce variability in patient selection and reported outcomes. Second, the majority of cited studies focus on Caucasian or Asian populations, limiting the generalizability of findings to other ethnic groups. Third, the literature on emerging therapies such as GLP-1 receptor agonists and SGLT-2 inhibitors is predominantly based on short-term trials with small sample sizes, precluding definitive conclusions regarding long-term efficacy and safety. Fourth, publication bias may exist, as studies reporting positive findings are more likely to be published than those with null or negative results. Finally, this is a narrative review rather than a systematic review or meta-analysis and therefore does not include a formal risk-of-bias assessment or quantitative synthesis of evidence. Future research should prioritize large, long-term, ethnically diverse randomized controlled trials to address these gaps.

Future therapeutic strategies may extend beyond conventional insulin sensitizers toward targeted modulation of neuroendocrine and steroidogenic pathways. Experimental agents including kisspeptin agonists (MVT-602), neurokinin-3 receptor antagonists such as fezolinetant, and AKR1C3 inhibitors have shown promising preliminary results for restoring ovulatory function, reducing androgen excess, and improving metabolic homeostasis. However, further clinical trials are required before these therapies can be incorporated into routine clinical practice.

## Conclusions

In summary, IR is a key driver of PCOS, contributing to reproductive, metabolic, and psychological complications. Early identification and comprehensive management, combining lifestyle modification with pharmacological interventions such as insulin sensitizers, are essential to improve outcomes and reduce long-term comorbidities. Addressing IR remains central to optimizing care for women with PCOS and guiding future therapeutic strategies.
